# Emergence of colistin-resistant *Enterobacter cloacae* and *Raoultella ornithinolytica* carrying the phosphoethanolamine transferase gene, *mcr-9*, derived from vegetables in Japan

**DOI:** 10.1128/spectrum.01063-23

**Published:** 2023-11-01

**Authors:** Christian Xedzro, Toshi Shimamoto, Liansheng Yu, Hui Zuo, Yo Sugawara, Motoyuki Sugai, Tadashi Shimamoto

**Affiliations:** 1 Laboratory of Food Microbiology and Hygiene, Graduate School of Integrated Sciences for Life, Hiroshima University, Higashihiroshima, Japan; 2 Antimicrobial Resistance Research Center, National Institute of Infectious Diseases, Higashimurayama, Japan; China Agricultural University, Beijing, China

**Keywords:** vegetables, *mcr-9*, *Enterobacteriaceae*, WGS, Japan

## Abstract

Colistin, a last-line antimicrobial agent, is recommended for the treatment of severe infections caused by multidrug-resistant (MDR) microorganisms. However, reports on plasmid-mediated mobilized colistin resistance (*mcr*) genes have prompted the importance of routine colistin resistance surveillance. Herein, we report the emergence of *Enterobacter cloacae* CST17-2 and *Raoultella ornithinolytica* CST129-1 carrying an *mcr-9.1* gene in vegetables from Japan. A total of 308 colistin-resistant isolates were retrieved from 200 fresh vegetables in Hiroshima Prefecture, Japan. PCR detection of *mcr-1* to *mcr-9* was conducted. While none of the isolates detected positive for *mcr-1* to *mcr-8* genes, we found two (0.65%) positive strains, *E. cloacae* CST17-2 and *R. ornithinolytica* CST129-1, that harbored *mcr-9.1* allele. These isolates were subjected to phenotypic susceptibility testing, whole-genome sequencing (WGS), PCR-based replicon typing, and conjugation experiment. We found that both isolates had high colistin resistance [minimum inhibitory concentration (MIC) 16 or >64 µg/mL] and showed MDR phenotypes. WGS of both isolates revealed *mcr-9* on a plasmid of the IncHI2/HI2A backbone. The *mcr-9*-bearing plasmid, pCST17-2_1, was self-transferable, although the pCST129-1_1 plasmid was not. Despite being colistin-resistant, the so-called two-component regulatory operon, *qseBC*, which induces polymyxin resistance, was absent from the genetic arrangements downstream of *mcr-9* in *R. ornithinolytica* CST129-1. Nonetheless, a conjugation experiment demonstrated that *mcr-9* in a *Raoultella*-type background is capable of mediating colistin resistance. *In silico* genomic analysis and comparison revealed distinct genetic structures surrounding *mcr-9*, especially in the downstream vicinities. The *E. cloacae* CST17-2 strain is of sequence-type ST738, a sequence type that has emerged in *mcr-9.1*-containing *E. cloacae*. Remarkably, we report the first *mcr-9*-carrying colistin-resistant *Enterobacteriaceae* isolated from Japanese vegetables, which is a grave public health concern. Our findings highlight the importance of strict epidemiological monitoring to track and/or prevent further dissemination of *mcr* homologs across the vegetable industry.

**IMPORTANCE:**

Plasmid-mediated mobile colistin-resistance genes have been recognized as a global threat because they jeopardize the efficacy of colistin in therapeutic practice. Here, we described the genetic features of two *mcr-9.1*-carrying Gram-negative bacteria with a colistin-resistant phenotype derived from vegetables in Japan. The colistin-resistant *mcr-9.1*, which has never been detected in vegetables, was located on a large plasmid in *Enterobacter cloacae* CST17-2 and *Raoultella ornithinolytica* CST129-1, suggesting a high chance of horizontal gene transfer. To the best of our knowledge, this is the first report of *mcr-9* in *R. ornithinolytica*. This study indicates that fresh vegetables might be a potential source for the transmission of *mcr-9* genes encoding resistance to frontline (colistin) and clinically relevant antimicrobials. The study also provides additional consideration for colistin use and the relevance of routine surveillance in epidemiological perspective to curb the continuous spread of *mcr* alleles.

## INTRODUCTION

Colistin (also called polymyxin E) is a cationic antimicrobial peptide discovered in Japan in 1947 (1). Since its commercialization in the 1950s, it has been considered as a miracle antibiotic because of its bactericidal efficacy against Gram-negative bacteria (GNB), including carbapenem-resistant *Enterobacterales* (2, 3). Although the effectiveness of colistin against GNB has not been questioned, previous intravenous administration has been coupled with side effects such as renal and neurological disorders (4). As a result, colistin was made readily unavailable in the 1980s in favor of antimicrobials with lower toxicity. The increasing incidence of carbapenem resistance among *Enterobacterales* triggered the reintroduction of colistin in the 2000s as a frontline antimicrobial in clinical practice (3, 5). Since then, clinicians have often administered colistin as a last-line antimicrobial defense against severe infections caused by multidrug-resistant (MDR) and extensively drug-resistant GNB (1, 6 – 8). In veterinary medicine, colistin is frequently used in pig, sheep, cattle, and poultry production, where it serves as a growth factor in animal feed and prevents the occurrence of enteric diseases (6).

Until 2015, bacterial resistance mechanisms against colistin were thought to be intrinsically driven, especially via specific chromosomal point mutations within two-component systems (TCSs), mainly PhoP/Q and PmrA/B, which are non-transferable through horizontal gene transfer (9, 10). However, the emergence of plasmid-located mobilized colistin resistance (*mcr*) has raised serious concerns regarding the continuous use of colistin and its significance in the spread of resistance, especially among *Enterobacterales*. In 2015, the first *mcr* gene, *mcr-1*, was identified in *Escherichia coli* in China (11). Following this report, a novel gene—*mcr-2*—was identified in porcine and bovine colistin-resistant *E. coli* isolates [minimum inhibitory concentration (MIC) 4–8 mg/L] that did not contain *mcr-1* in Belgium (12). Since then, epidemiological investigations have identified several *mcr* homologs, including *mcr-3* (13), *mcr-4* (14), *mcr-5* (9), *mcr-6* (15), *mcr-7* (16), *mcr-8* (17), and *mcr-9* (18). Recently, a novel *mcr-10* was identified in *Enterobacter roggenkampii* and was added to the documented catalogs of *mcr* homologs (19). This suggests that either plasmid-borne *mcr* genes existed long ago or that horizontal gene transfer played an important role in the rapid dissemination of such *mcr* homologs. *mcr* encodes phosphoethanolamine transferase, which mediates colistin resistance by modifying the lipid A moiety of lipopolysaccharide, resulting in the reduction of negative charges across bacterial membrane (2, 19, 20).

Although colistin is largely used as an in-feed growth factor in animal production, *mcr*-producing bacteria have also been identified in animal sources and environmental samples, including sewage and river water (21, 22). Recently, fresh farm produce—including vegetables—has gained much attention because of the reported incidence and outbreaks of foodborne bacteria carrying antimicrobial resistance genes (23 – 25). Given that vegetables are mostly consumed either raw or partially processed, ingesting critically important foodborne pathogens is possible, which is of great concern. This increases the chance of human exposure to antimicrobial-resistant bacteria, including *mcr*-carrying strains; specifically, this represents a risk factor for consumer safety. Of note, the presence of *mcr*-carrying bacteria has been documented within vegetables. To date, eight studies have identified vegetable-sourced *mcr*-producing GNB (23). For example, *mcr-1* was detected in *Enterobacteriaceae* isolated from two vegetables imported to Switzerland (21). Additionally, an *mcr-1*-carrying *E. coli* strain was recovered from lettuce in South Korea during a national surveillance in 2018 (26). In Portugal, two *E. coli* isolates carrying *mcr-1* were isolated from domestic lettuce (27, 28). The remaining reports describe *mcr-1*-harboring *Enterobacteriaceae* identified in vegetables in China (8, 25, 29, 30). Very recently, MDR *Enterobacter hormaechei* carrying the novel *mcr-9* was isolated for the first time from vegetables in the USA (31). To the best of our knowledge, no previous study has identified *mcr-9*-carrying *Enterobacter cloacae* and *Raoultella ornithinolytica* with a colistin-resistant phenotype in vegetables. It was previously found that the two-component regulatory operon, *qseBC*, induces *mcr-9* expression, leading to colistin resistance (32). Other studies tested 30 *mcr-9*-carrying *Enterobacteriaceae* and found that those isolates were susceptible to colistin and lacked the *qseBC* regulatory system (33). Further studies reported colistin resistance in isolates lacking such regulatory operon (34, 35), indicating uncertainties as to what phenotype *mcr-9* gene may carry. Hence, genomic epidemiological surveillance investigations will be useful in providing updated information on *mcr-9* and the emergence of new resistance traits within the food chain and vegetable production continuum.

Considering that vegetables are part of a healthy diet and the increasing prevalence and wide dissemination of *mcr* genes have been reported, it is of the utmost urgency to investigate the occurrence of *mcr*-positive bacteria in vegetables in Japan. In a routine, prefectural surveillance to track the changes and the prevalence of antimicrobial resistance determinants among different local ecosystems (river water, sewage, meat, soil, and vegetables), our research group retrieved colistin-resistant GNB bacterial isolates (MIC >2 µg/mL) from several vegetable samples collected in Hiroshima Prefecture, Japan. Although the use of colistin as a feed additive for food-producing animals has been abolished in Japan since 2018, its therapeutic administration to cattle and pigs has been allowed (36). Given that the current reports on *mcr* genes in Japan are mostly linked to *E. coli* and *Salmonella enterica* derived from livestock, we speculated that the discharge of wastewater and sewage irrigation from animal farms might transfer colistin-resistant bacteria to vegetables. We then screened our collection of colistin-resistant isolates for the presence of *mcr-1* to *mcr-9*. Moreover, it is worth noting that there are no reports describing the emergence of *mcr-9*-carrying *Enterobacteriaceae* in vegetables, apart from the recently reported *E. hormaechei* in the USA (31). Therefore, in this study, we characterized the genetic features of vegetable-derived plasmid-borne *mcr-9.1* in colistin-resistant *E. cloacae* and *R. ornithinolytica* during a prefectural epidemiological or antimicrobial surveillance in Japan by whole-genome sequencing (WGS).

## RESULTS

### Screening for *mcr-1* to *mcr-9* and strain identification

During a One Health antimicrobial surveillance from June 2020 to December 2021, our research group recovered 308 colistin-resistant (MIC >2 µg/mL) GNB isolates from 200 fresh vegetable samples randomly collected in Hiroshima Prefecture, Japan. We have screened the isolates for the presence of *mcr-1* to *mcr-9*. While none of the isolates detected positive for *mcr-1* to *mcr-8* genes, we found two (0.65%) positive strains, *E. cloacae* CST17-2 and *R. ornithinolytica* CST129-1 that carried the recently reported putative phosphoethanolamine transferase gene, *mcr-9* (18). *E. cloacae* CST17-2 was isolated from a bean sprout (a common ingredient in many Asian cuisines), while *R. ornithinolytica* CST129-1 was an isolate from myoga (Japanese ginger) (Table 1). They were identified by amplifying and sequencing the 16S rRNA fragments and confirmed by WGS.

**TABLE 1:** Sample information and antimicrobial resistance profiles of *mcr-9*-carrying colistin-resistant *Enterobacteriaceae* isolates within this study^*a*^

Strain	Vegetable sample	Identification/species	Year of isolation	Prefecture	Resistance phenotypes
CTS17-2	Bean sprout	*E. cloacae*	2020	Hiroshima	AMP, CTX, CAZ, FOX, CST, FOF
CST129-1	Myoga	*R. ornithinolytica*	2021	Hiroshima	AMP, CST, FOF

^*a*^ AMP, ampicillin; CAZ, ceftazidime; CST, colistin; CTX, cefotaxime; FOF, fosfomycin; FOX, cefoxitin.

### Antimicrobial susceptibility

Susceptibility testing by the broth microdilution method showed that *E. cloacae* CST17-2 and *R. ornithinolytica* CST129-1 specified robust resistance to colistin with MIC > 64 µg/mL and 16 µg/mL, respectively. Additional testing revealed that *E. cloacae* CST17-2 exhibited resistance to various antimicrobials, including ampicillin (MIC > 512 µg/mL), cefotaxime (MIC 8 µg/mL), ceftazidime (MIC 32 µg/mL), cefoxitin (MIC > 128 µg/mL), and fosfomycin (MIC > 512 µg/mL) (Table 2). We also found that *R. ornithinolytica* CST129-1 conferred additional resistance to ampicillin and fosfomycin (MIC 512 µg/mL or >512 µg/mL) (Table 2).

**TABLE 2:** MIC of various antimicrobials for *mcr-9*-carrying *Enterobacteriaceae* and their transconjugants^*d*^

Antibiotics	MIC (µg/mL)^*a*^					
	*E. cloacae* CST17-2	*E. coli* J53 + pCST17-2_1^*b*^	*R. ornithinolytica* CST129-1	*E. coli* J53 *+* pCST129-1_1^*c*^	*E. coli* J53	*E. coli* ATCC 29522
Ampicillin	**>512**	**>512**	**512**	–	8	2
Cefotaxime	**8**	**32**	1	–	0.25	0.25
Ceftazidime	**32**	**64**	2	–	1	1
Cefoxitin	**>128**	**128**	4	–	8	4
Meropenem	1	1	1	–	<0.0625	<0.0625
Kanamycin	4	8	4	–	8	8
Gentamicin	1	1	0.5	–	4	4
Chloramphenicol	8	8	2	–	8	4
Ciprofloxacin	<0.125	<0.125	<0.125	–	<0.25	<0.25
Tetracycline	4	4	2	–	2	1
Colistin	**>64**	**>64**	**16**	–	0.5	0.5
Fosfomycin	**>512**	ND	**>512**	–	8	8

^*a*^ MIC interpretation was performed according to CLSI and EUCAST recommendations (for colistin). The resistance results are shown in bold font.

^*b*^ *E. coli* J53 + pCST17-2_1: transconjugant.

^*c*^ Transconjugants were not obtained.

^*d*^ pCST17-2_1: plasmid of *E. cloacae* carrying *mcr-9*. pCST129-1_1: plasmid of *R. ornithinolytica* carrying *mcr-9*. ND, not determined.

### Genome characterization of *mcr-9*-carrying *Enterobacteriaceae*

A hybrid assembly of both Illumina MiSeq short reads and Oxford Nanopore long reads revealed the complete circular chromosome and plasmids contained in each strain. The chromosome of the *E. cloacae* CST17-2 strain was 4,819,105 bp in size, with an average guanine-cytosine (GC) content of 55% and harbored *fosA* (a gene encoding fosfomycin resistance) and *bla*_ACT_ (a chromosomal AmpC gene in *Enterobacter* that renders resistance to penicillins and cephalosporins) genotype (Table 3). Species identification based on average nucleotide identity (ANI, a metric for comparing genetic relatedness or to verify taxonomic identities) analysis and the k-mer algorithm identified the strain as *E. cloacae*, as it shared 99.95% sequence identity with the reference strain for *E. cloacae* A1137 (accession number NZ_CP021851.1), with a query coverage of 91%; given this, it was designated as *E. cloacae* CST17-2. Further analysis of the WGS data indicated that the strain was of sequence type ST738, a sequence type that has emerged in *mcr-9.1-*carrying *E. cloacae*.

**TABLE 3:** Genomic features of chromosomes and plasmids identified in *mcr-9*-carrying colistin-resistant *E. cloacae* and *R. ornithinolytica^a^*

Species	Chromosome/plasmid	Length (bp)	GC %	MLST/pMLST	Incompatibility group	Resistance genes	Tellurium ion resistance genes
*E. cloacae*	Chromosome	4,819,105	55	ST738	–	*fosA*, *bla*_ACT_	ND
	pCST17-2_1	304,688	46	DLST2	IncHI2/HI2A	*mcr-9*	*ter* (*A*, *C*, *D*, *E*, *F*)
	pCST17-2_2	5,113	52	Unknown	ND	ND	ND
	pCST17-2_3	4,665	51	Unknown	ND	ND	ND
	pCST17-2_4	4,305	54	Unknown	Col440II	ND	ND
*R. ornithinolytica*	Chromosome	5,451,172	56	–	–	*fosA*, *bla*_PLA-1a_	ND
	pCST129-1_1	306,744	48	DLST1	IncHI2/HI2A	*mcr-9*	*ter* (*A*, *C*, *D*, *E*, *F*)
	pCST129-1_2	134,923	52	Unknown	ND	ND	ND
	pCST129-1_3	5,215	51	Unknown	ND	ND	ND
	pCST129-1_4	4,155	44	Unknown	Col440I	ND	ND

^*a*^ ND, not detected.

The *R. ornithinolytica* CST129-1 strain was confirmed as *R. ornithinolytica* because it had ANI value of 99.62% with the reference strain for *R. ornithinolytica* Yangling I2 (83% query coverage; GenBank accession number NZ_CP013338.1) identified in China. The chromosome size was 5,451,172 bp, with 56% GC content, and carried two known antimicrobial resistance genes, *bla*_PLA-1a_ and *fosA* (Table 3).

### Detection of *mcr-9*-associated plasmids and *in silico* molecular features

**Fig 1:**
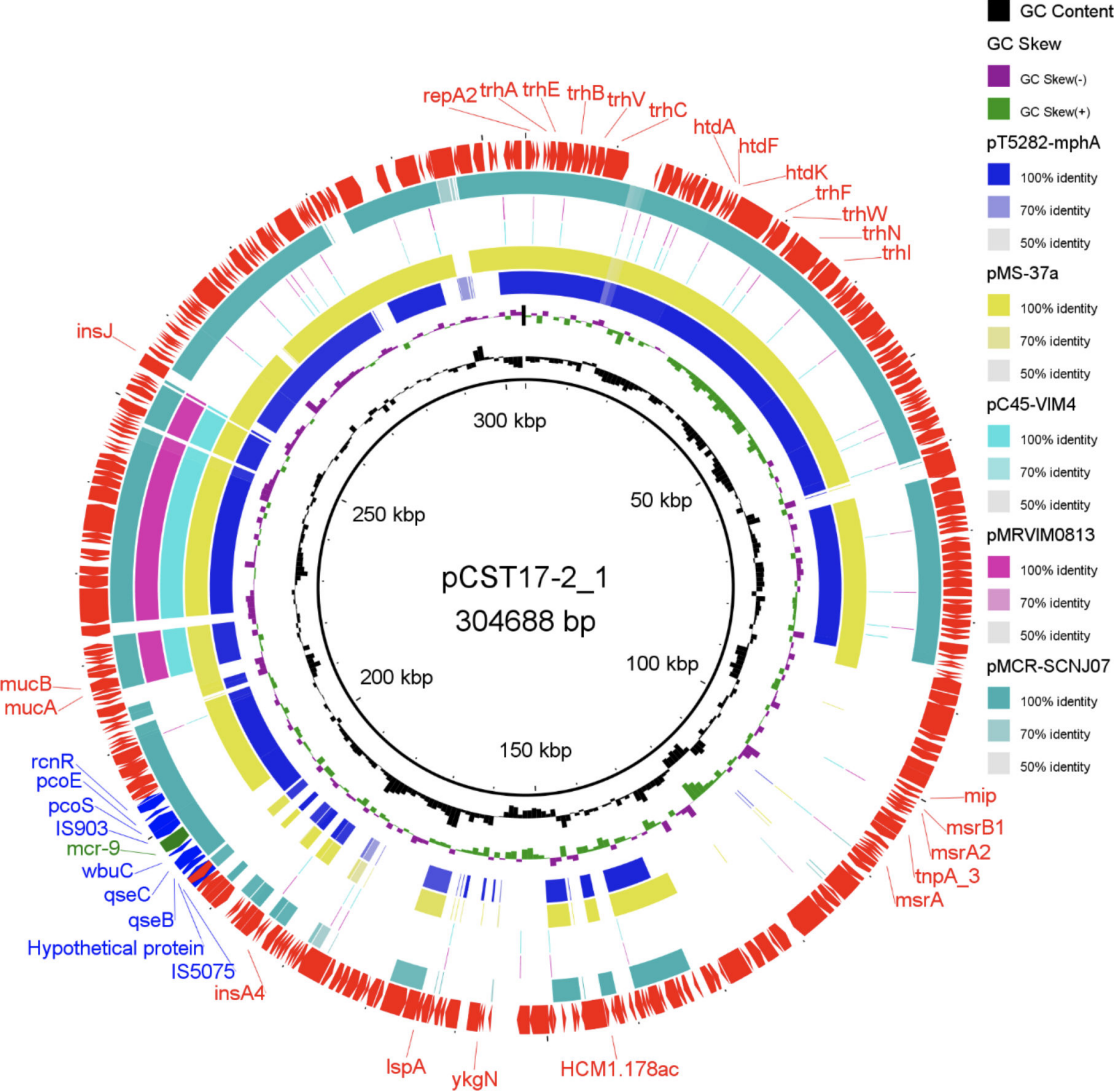
A circular map of *mcr-9*-carrying HI2 plasmids. The complete sequence of pCST17-2_1 identified in this study was used as a reference. Genes and open reading frames (ORFs) of the reference plasmid are shown with arrowheads indicating their transcriptional orientations (outer ring). The plasmids were included in the following order from the inner ring: pCST17-2_1 (this study), pT5282-mphA (KY270852), pMS-37a (CP053191), pC45-VIM4 (LT991958), pMRVIM0813 (KP975077), and pMCR-SCNJ07 (NZ_MK933279.1).

**Fig 2:**
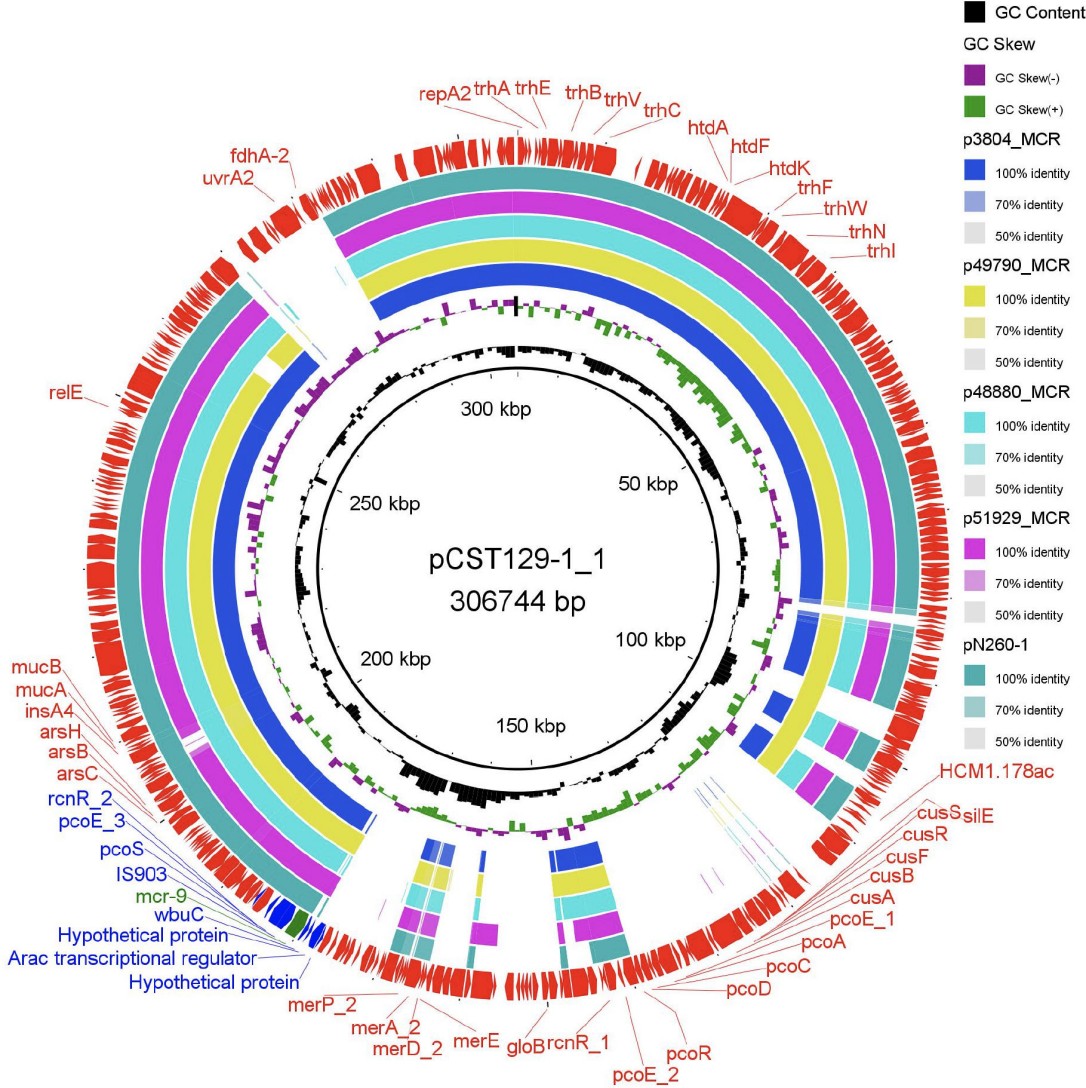
A structural comparison between *mcr-9*-carrying HI2 plasmids. The complete sequence of pCST129-1_1 identified in this study was used as a reference. The outer ring represents the genes and open reading frames (ORFs) with arrowheads indicating their transcriptional orientations. The plasmids were included in the following order from the inner ring: pCST129-1_1 (this study), p3804_MCR (NZ_CP064657.1), p49790_MCR (NZ_CP059425.1), p48880_MCR (NZ_CP059421.1), p51929_MCR_VIM (CP059429.1), and pN260-1 (NZ_AP023448.1).

To determine the location of *mcr-9*, we analyzed the *de novo* assembled genomes obtained from both strains. Bioinformatic analysis using PlasmidFinder detected four plasmids, including Col440II/I-type plasmids within each strain, with sizes ranging from 4,155 to 306,744 bp (Table 3). *E. cloacae* CST17-2 has a 304,688-bp plasmid of IncHI2/HI2A backbone, designated as pCST17-2_1. This plasmid has a GC content of 46% (Table 3; Fig. 1). Coincidentally, the same plasmid backbone (IncHI2/HI2A), designated as pCST129-1_1 (GC content, 48%), was identified in *R. ornithinolytica* CST129-1 and was 306,744 bp in size (Table 3; Fig. 2). When compared to each other, both plasmids shared ~98.51% sequence identity (~64% query coverage) as detected by pairwise genome comparison using JSpeciesWS (37). Additionally, *in silico* DNA-DNA hybridization based on Genome-to-Genome Distance Calculator (GGDC) (38) revealed a high similarity score of 94.55%, indicating a high correlation between the two plasmids. Furthermore, full sequence queries of the two plasmids by BLAST showed that they were similar to a portion of IncHI2 plasmid identified in *Serratia marcescens* (~99.73% sequence identity and ~75% query coverage; GenBank accession number BX664015). PCR-based replicon typing confirmed the presence of IncHI2 plasmids, as suggested by the WGS data.

Plasmid multilocus sequence type (pMLST) analysis confirmed that pCST17-2_1 and pCST129-1_1 belong to the double locus sequence types DLST2 and DSLT1, respectively (Table 3). *In silico* analysis of both plasmids revealed a 1,620-bp putative phosphoethanolamine transferase encoding *mcr-9.1* gene (539 aa), which exhibited >99.90% nucleotide sequence identity and 100% coverage with a similar *mcr-9* gene identified in *S. enterica* subsp. *enterica* serovar Typhimurium (GenBank accession number NZ_NAAN01000063.1). The plasmids do not carry any other antibiotic resistance determinants but rather possess a variety of tellurium ion resistance genes and other metal ion resistance genes. We then performed a circular comparison of the IncHI2 plasmids identified within this study alongside similar plasmids downloaded from GenBank using BLAST Ring Image Generator (BRIG) (39) (Fig. 1 and 2). A pairwise genome comparison using JSpeciesWS (37) showed that plasmid pCST17-2_1 has homologies with pT5282-mphA (97.46% ANI), pMS-37a (97.81% ANI), pC45-VIM4 (98.57% ANI), pMRVIM0813 (98.57% ANI), and pMCR-SCNJ07 (97.71% ANI) (Fig. 1). Additionally, comparative analyses revealed that plasmid pCST129-1_1 also has homologies with p3804_MCR (99.41% ANI), p49790_MCR (99.30% ANI), p48880_MCR 99.23% ANI), p51929_MCR_VIM (99.33% ANI), and pN260-1 (99.40% ANI) (Fig. 2) downloaded from National Center for Biotechnology Information (NCBI) databank.

### Genomic structure surrounding the *mcr-9* gene

**Fig 3:**
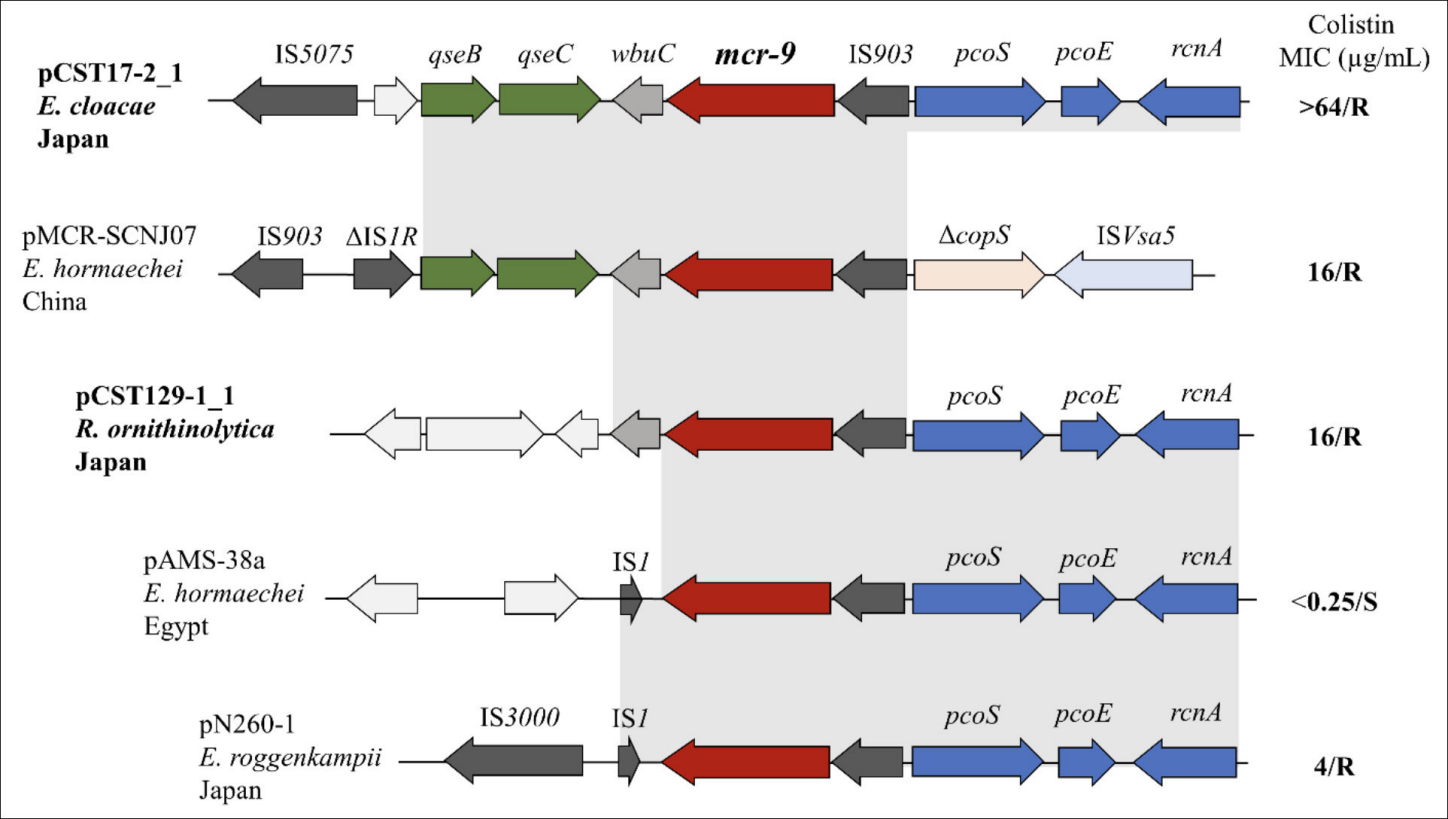
The genetic context of *mcr-9*. Comparative analysis between the genetic environments of *mcr-9* identified in this study with other *mcr-9*-carrying HI2 plasmids is shown. Different genetic structures were identified, with intact IS903 located at the immediate upstream regions of all the *mcr-9* genes. The downstream vicinities are different for each plasmid. *mcr-9* genes are indicated with red arrows. Regions of homology (~99.90% sequence similarity as determined by pairwise alignment of those regions) are highlighted in gray rectangles. Arrows indicate the positions and directions of the transcriptional orientations of the genes. The colistin MICs of those isolates are indicated next to each plasmid where R is resistant and S means susceptible. NCBI GenBank accession numbers are as follows: pCST17-2_1 (this study), pMCR-SCNJ07 (NZ_MK933279.1), pCST129-1_1 (this study), pAMS-38a (NZ_CP051133.1), and pN260-1 (NZ_AP023448.1).

Genome visualization of *mcr-9*-associated plasmids revealed different genetic orientations. Analysis of the genetic structure of pCST17-2_1 showed that *mcr-9* was enclosed by two intact insertion sequences (ISs), IS903 (IS5 family) and IS5075 (IS110 family) located at the upstream and downstream boundaries, respectively. Within this region, a hypothetical protein, *wbuC* (encoding a functionless putative cupin metalloprotein), was located downstream, followed by a two-component regulatory system consisting of a sensor histidine kinase encoding *qseC* and a response regulator *qseB* genes (Fig. 3). The presence of similar downstream proteins induces colistin resistance in *Enterobacteriaceae* as previously reported (40, 41). The IS5075-*qseB-qseC-wbuC-mcr-9*-IS*903* operon was in an arrangement similar to that found within multiple genera of *Enterobacteriaceae*, including *E. hormaechei* (NZ_MK933279.1), *E. coli* (GCA_900500325), *S. enterica* (CP026661.1), *E. cloacae* (NZ_MF344583), and *Leclercia adecarboxylata* (MH909331.1). Unlike *E. cloacae* CST17-2, we observed a different genetic disposition for *R. ornithinolytica* CST129-1, which was characterized by a lack of downstream *qseB*-like and *qseC*-like regulatory operons (Fig. 3). The fact that *mcr-9* conferred colistin resistance, despite the absence of the *qseB*/*qseC* system, brings new insights to the idea that *mcr-9* may be activated by other proteins from *R. ornithinolytica* or that resistance is mediated by an unknown mechanism.

### Transferability of *mcr-9*-harboring plasmid

To better understand whether the plasmids carrying *mcr-9* could naturally restore colistin resistance in heterologous bacterial hosts, we conducted a series of conjugation experiments using multiple recipient backgrounds. Interestingly, *E. cloacae* CST17-2 successfully transferred its resistance plasmid to *E. coli* J53 and conferred colistin resistance, indicating the self-transferability of this plasmid (Table 2).

Despite several attempts in conjugation between *R. ornithinolytica* CST129-1 and *E. coli* J53, transconjugants carrying *mcr-9* were not obtained (Table 2). We repeated this process using two strains of *R. ornithinolytica* (MA-1-1-r and CAA-1-5-r) with different resistance phenotypes as recipients. Both strains were isolated from a sewage effluent (discharged from Asahi-cho Sewage Workstation, Hiroshima City) on 11 May 2021 in the same region and tested susceptible to colistin (MIC ≤ 1µg/mL). Plasmid transfer in this context was also unsuccessful even at varying conjugation temperatures of 25°C, 30°C, and 37°C (Table 4). We then speculated that *mcr-9* may be activated by other proteins within *Raoultella* spp. In order to verify what function *Raoultella* background has on *mcr-9* expression and considering the fact that *Raoultella* is an *Enterobacteriaceae* closely related to *Klebsiella* (42), we further included a colistin-susceptible (MIC, 0.125 µg/mL) *Klebsiella pneumoniae* LM22-1 (accession number DRA009528) (43) as a donor (Table 4). *K. pneumoniae* LM22-1 carries *mcr-9* on a 281,252-bp plasmid of IncHI2/HI2A backbone, which is in a cassette with a similar genetic context as *R. ornithinolytica* CST129-1. Interestingly, *K. pneumoniae* transferred its colistin-susceptible *mcr-9*-carrying plasmid to both *R. ornithinolytica* (MA-1-1-r and CAA-1-5-r) recipients. When determining colistin MIC for the *Raoultella* transconjugants (carrying the *Klebsiella* plasmid), we noticed a remarkable increase in MIC (4‒32 µg/mL) (Table 5), suggesting that *mcr-9* was highly expressed in a *Raoultella-*type background, which supported our hypothesis. This indicated that unknown proteins originating from *Raoultella* spp. might be potential players in *mcr-9* activation, leading to colistin resistance. We then aligned the amino acid sequences of *mcr-9* derived from *E. cloacae* CST17-2 and *R. ornithinolytica* CST129-1 with that of *K. pneumoniae* LM22-1. We found a mutational event characterized by a single amino acid deletion (539 vs. 540 aa) in the aligned sequences of *mcr-9* compared to that found in *K. pneumoniae* LM22-1 (Fig. S1), further confirming *mcr-9.1* identified in this study. It is important to note that *mcr-9.1* is characterized by 539 aa.

**TABLE 4:** Plasmid transfer success of *mcr-9*-carrying *R. ornithinolytica* CST129-1 and *K. pneumoniae* LM22-1 after conjugation experiment^*a*^

Expected transconjugants	Agar plate for selecting transconjugants	Plasmid transfer		
		25°C	30°C	37°C
MA-1-1-r + pCST129-1_1	LB + 16-µg/mL CHL and 4-µg/mL CST	Not transferred	Not transferred	Not transferred
CAA-1-5-r + pCST129-1_1	LB + 20-µg/mL TET and 4-µg/mL CST	Not transferred	Not transferred	Not transferred
MA-1-1-r + pLM22-1-VIM-1	LB + 16-µg/mL CHL and 2-µg/mL MEM	Transferred	Transferred	Transferred
CAA-1-5-r + pLM22-1-VIM-1	LB + 20-µg/mL TET and 2-µg/mL MEM	Transferred	Transferred	Transferred

^*a*^ pLM22-1-VIM-1: plasmid of *K. pneumoniae* LM22-1 carrying *mcr-9* and *bla*_VIM-1_ (this plasmid was co-transferred with plasmid pLM22-1-NDM-1 identified in the same strain). MA-1-1-r and CAA-1-5-r are *R. ornithinolytica* strains that were used as recipients. CHL, chloramphenicol; CST, colistin; MEM, meropenem; TET, tetracycline.

**TABLE 5:** MIC of antimicrobials for *Raoultella* transconjugants and *K. pneumoniae* LM22-1^*b*^

Antimicrobials	*K. pneumoniae* LM22-1 (donor)^*a*^	MA-1–1-r (recipient)	MA-1–1-r *+* pLM22-1-VIM-1	CAA-1–5-r (recipient)	CAA-1–5-r *+* pLM22-1-VIM-1
Ampicillin	ND	**>512**	**>512**	**>512**	ND
Cefotaxime	**512**	0.5	**>32**	0.5	**>32**
Ceftazidime	ND	1	**>128**	**>128**	ND
Cefoxitin	ND	8	**>128**	**32**	ND
Meropenem	**16**	0.5	**32**	0.25	ND
Kanamycin	**256**	4	**>128**	**128**	ND
Gentamicin	4	0.5	**16**	1	ND
Chloramphenicol	4	**128**	**>128**	4	ND
Ciprofloxacin	1	1	**16**	0.5	ND
Tetracycline	0.5	**128**	**8**	**128**	**64**‒**128**
Colistin	0.125	0.5‒1	**16‒32**	1	**4**
Fosfomycin	**>1,024**	**>512**	ND	**>512**	ND

^*a*^ MICs were adapted from the published article (refer to the published article on *K. pneumoniae* LM22-1, accession number DRA009528). The resistance results are shown in bold font.

^*b*^ ND, MIC not determined. MA-1-1-r + pLM22-1-VIM-1: transconjugant (contains plasmid pLM22-1-NDM-1 identified in *K. pneumoniae* LM22-1). CAA-1-5-r + pLM22-1-VIM-1: transconjugant (contains plasmid pLM22-1-NDM-1 identified in *K. pneumoniae* LM22-1). MA-1-1-r and CAA-1-5-r are *R. ornithinolytica* strains with different resistance phenotypes.

## DISCUSSION

MDR organisms, including colistin- and carbapenem-resistant *Enterobacterales*, have been recognized as an unprecedented global concern (19). Currently, one of the most worrisome issues pertains to the frequent occurrence of plasmid-borne *mcr* alleles, resulting in the reduced effectiveness of colistin, one of the few therapeutic options for treating severe MDR infections. In fact, the increasing prevalence of the novel putative phosphoethanolamine transferase gene, *mcr-9*, among colistin-resistant *Enterobacteriaceae* is of growing concern and indicates a high propensity for poor clinical outcomes in the future because its mechanism of resistance is still unclear.

*mcr-9* has been found in multiple bacterial species, including *S. enterica* (44, 45), *E. coli* (32, 45), *E. hormaechei* (40, 46), *Enterobacter kobei*/*Enterobacter asburiae* (47), and *K. pneumoniae* (43), and is mostly hosted in the *E. cloacae* complex isolated from humans and animals (48). Understanding the transmission routes and mechanisms of resistance is crucial for making tailored decisions to controlling the spread of *mcr* alleles. To the best of our knowledge, the detection of *mcr-9* in MDR *Enterobacteriaceae* isolated from vegetables has been reported only once (31). Therefore, vegetables might be a potential transmission route for *mcr-9*-producing MDR bacteria.

In the present study, we report the first genomic characterization of plasmid-borne colistin-resistant *E. cloacae* and *R. ornithinolytica* carrying *mcr-9* from fresh vegetables in Japan. Additionally, we reported the emergence of *mcr-9* in *R. ornithinolytica*, an *Enterobacteriaceae* closely related to *Klebsiella* spp. *R. ornithinolytica* is often isolated from aquatic and soil environments and has been found to be associated with community-acquired infection (8). Susceptibility testing indicated that both isolates exhibited MDR phenotypes and mediated colistin resistance (MIC 16 or >64 µg/mL), which were remarkably higher than that previously reported in *mcr-9*-carrying *S*. Typhimurium (18). The *mcr-9* in this *Salmonella* strain was characterized by 64.5% amino acid identity to *mcr-3* and was naturally susceptible to colistin (MIC 0.25–0.5 µg/mL). It has previously been shown that a two-component regulatory system, *qseBC*, induces *mcr-9* expression, leading to colistin resistance (32). Genomic analysis of *E. cloacae* CST17-2 revealed a functional two-component regulatory system encoding *qseBC* genes located downstream of *mcr-9*, further supporting the role of these tandem genes in inducing colistin resistance (Fig. 3). Such genetic arrangements have previously been identified in *mcr-9*-carrying colistin-resistant *S. enterica* strains in Korea (44) and China (40). The fact that *mcr-9* is not as concerning as other *mcr* homologs (37), from a public health perspective, should be reconsidered, as epidemiological investigations and susceptibility testing continue to reveal different emerging resistance phenomena related to this gene. It appears that the changing genetic disposition associated with *mcr-9* continues to facilitate its effect on colistin, resulting in reduced susceptibility patterns among *Enterobacterales*.

Surprisingly, contrary to other studies that reported colistin susceptibility among *Enterobacteriaceae* lacking chromosomal or plasmid *qseBC* from Egypt (49), Lebanon (46), and Japan (43), we have found a unique colistin-resistant phenotype (MIC 16 µg/mL) within *R. ornithinolytica* CST129-1, although the functional *qseBC*-like cassette responsible for inducing colistin resistance among *Enterobacterales* was absent from the genetic arrangements downstream of *mcr-9*. Since no functional mutations were detected on the *R. ornithinolytica* CST129-1 chromosome, we speculated that this resistance phenotype was suggestive of an unknown mechanism or that other proteins in *Raoultella* might have activated *mcr-9* expression, leading to colistin resistance. Although conjugation failed to transfer pCST129-1_1 to *E. coli* J53 and the two *R. ornithinolytica* (MA-1-1-r and CAA1-5-r) recipients, we proved our assumption in another conjugation assay by using a colistin-susceptible *mcr-9*-carrying *K. pneumoniae* LM22-1 as a donor to *R. ornithinolytica* (MA-1-1-r and CAA1-5-r). In this context, we found that *mcr-9* was highly expressed in the transconjugants, leading to a remarkable increase in colistin MIC levels, further supporting the role *mcr-9*-carrying *Raoultella* spp. may play in mediating colistin resistance among the *Enterobacteriaceae*.

Multiple *Enterobacterial* species, including *Salmonella* (45), *E. coli* (45), *E. asburiae* (50), *Citrobacter telavivum* (51), and *E. hormaechei* (52) with the so-called *qseBC* genes, have been reported to exhibit colistin-susceptible phenotypes. In contrast, some other studies also found that lack of such a tandem system in certain bacterial species was associated with colistin-resistant phenotypes, as with *E. roggenkampii* (34), *E. cloacae* complex (48), and *S*. Newport (35). These observations suggest that the mechanisms leading to a reduced susceptibility to polymyxin are more related to other factors (not mentioning chromosomal point mutations), in which a different strain background may be a contributing factor underlying the mystery behind *mcr-9* expression. As a matter of growing concern, though *mcr-9* expression is governed by a regulatory system, it may not necessarily be associated with colistin susceptibility in the absence of plasmid-borne *qseBC* genes as previously reported.

Evidence suggests that heavy metal ions impose a selective pressure favoring the co-selection of metal and antibiotic resistance genes (53 – 55). Two main mechanisms have been reported to govern this process. One is co-resistance, in which genes with specific resistance phenotypes co-exist on the same mobile genetic element (i.e., plasmid or transposons), and the second is cross-resistance, where bacteria can develop resistance to both antibiotics and heavy metals at the same time (54, 56). It appears that co-selection is a mechanism of drug resistance because transcriptional and translational responses related to metal ions and antibiotics are genetically linked, in which bacteria exposed to heavy metals are simultaneously resistant to metals and antibiotics (54, 57, 58). Considering that the genetic context of *mcr-9* consists of copper-binding proteins and metal efflux systems, we postulate that the presence of plasmid genes encoding metal ion resistance could be potential players in the induction of a positive feedback response leading to *mcr-9* expression and consequently mediating colistin resistance. However, further studies are required to draw definitive conclusions regarding this speculation. Very recently, a different phenomenon of colistin resistance was reported in a Japanese research laboratory, in which *E. asburiae* strain carrying chromosomal *mcr-9* coupled with *qseBC* showed susceptibility to colistin (MIC 0.5 µg/mL) in a static culture incubation using broth microdilution but did grow at 8 µg/mL in a shaking culture incubation (59). In contrast, a remarkable increase in colistin MIC was observed when the standardized cation-adjusted Muller-Hinton broth was amended with casein, peptone, and tryptone, further supporting the argument that several other factors within the immediate environment might be responsible for *mcr-9* expression. It is also worth mentioning that the observed colistin MIC for this isolate was relatively higher (32–128 µg/mL) compared to those lacking the regulatory operon, which is supportive of the functional role of the *qseBC* tandem system.

Further analysis of the genetic context surrounding *mcr-9* revealed genetic structures and arrangements distinct from those previously reported (34, 40, 49). However, a similar structure exists between the *E. cloacae* CST17-2 strain identified in this study and the *E. hormaechei* strain reported in China (40), in which *mcr-9* was bracketed by two ISs (Fig. 3). These mobile elements facilitate the mobilization of resistance genes in different plasmids. Considering that *mcr-9* and the two-component regulatory operons were identified within these flanking vicinities, they indicated the potential for being carried along during such mobilization and transposition events. We observed that the upstream regions of *mcr-9* within the *R. ornithinolytica* CST129-1 strain were homologous to pAMS-38a (NZ_CP051133.1) and pN260-1 (NZ_AP023448.1) reported in Egypt (49) and Japan (34), respectively, but displayed different orientations downstream. Collectively, these insights suggest that clinical infections due to *mcr-9*-carrying strains would be problematic when treated with colistin since some bacterial species in this context seem to have elevated colistin MIC beyond the steady-state plasma concentration of 2 µg/mL (60).

WGS analysis showed that both isolates harbored *mcr-9* on a large DLST HI2/HI2A plasmid (>300,000 bp). No other determinants of antibiotic resistance were detected on these plasmids. Comparative genome analysis revealed that these plasmids shared homologies with similar *mcr-9*-carrying HI2 plasmids downloaded from public database (Fig. 1 and 2). We then conducted conjugation assays to investigate the transferability of these epidemic plasmids and the expression of *mcr-9* in heterologous bacterial hosts. Transconjugants with high colistin MIC were obtained when conjugating pCST17-2_1, indicating that *mcr-9* was located on a self-transferable plasmid (Table 2). This observation is consistent with previous reports that the worldwide dissemination of *mcr-9* is mainly driven by HI2/HI2A plasmids (18, 48, 49). Furthermore, similar conjugative *mcr-9*-carring plasmids have been found in food and clinical isolates in Japan (34, 43, 61), suggesting a One Health perspective on the transmission of such *mcr* homolog. Either the fact that *R. ornithinolytica* cannot conjugate was attributed to a lack of conjugative transfer mechanisms or the fact that the genetic backgrounds of the recipients were not suitable for this specific plasmid. Evidence suggests that most very large plasmids tend to be less mobilizable (62); hence, successful conjugation may not be easily achieved in certain bacterial species.

The *E. cloacae* CST17-2 strain belongs to the sequence type ST738. A search within NCBI database revealed 386 *E. cloacae* genomes; however, ST738 was not found in *mcr-9*-containing strains, suggesting that this ST type has emerged in *mcr-9.1*-carrying *E. cloacae*. It is worth mentioning that *mcr-9* was previously reported to have originated from a yet-to-identify bacterial species that is closely related *Buttiauxella* spp. (19), given that this might relate to the uncertainties as to what function this *mcr* allele may carry. Further studies will be conducted to elucidate other mechanisms, if any, that may be associated with *mcr-9* expression.

In conclusion, our findings indicate that fresh vegetables constitute a possible transmission route for the dissemination of colistin-resistant genes among *Enterobacterales*. The detection of highly colistin-resistant MDR strains in food sources is frustrating and poses a food safety risk because these strains can be transmitted to humans. Here, we described the first complete genomic study of the characteristics of colistin-resistant *mcr-9.1*-carrying *Enterobacteriaceae* isolated from fresh vegetables in Japan. To the best of our knowledge, this is the first report of foodborne *R. ornithinolytica* carrying *mcr-9*. Further research, including extensive epidemiological investigations and continuous susceptibility testing, is required to assess the occurrence of novel resistance traits in all prefectures of Japan and other countries worldwide. It is equally important to investigate other unknown mechanisms that lead to reduced susceptibility to colistin both *in vitro* and *in vivo*.

## MATERIALS AND METHODS

### Bacterial isolation and total genomic DNA extraction

During the period from June 2020 to December 2021, 200 fresh vegetables were purchased from popular supermarkets in Hiroshima Prefecture, Japan. Bacterial isolation was as follows: briefly, a 25-g portion of each vegetable sample was homogenized in 225 mL of buffered peptone water (Nissui Pharmaceutical Co., Ltd., Tokyo, Japan) using a stomacher blender for 1 min. The resulting suspensions were directly plated on MacConkey agar (Eiken Chemical Co., Ltd., Tochigi, Japan) containing 2 µg/mL of colistin (Wako Pure Chemical Industries, Ltd., Osaka, Japan), and the plates were incubated at 37°C for 24 h. After incubation, bacterial colonies were preferentially selected and re-cultured on similar antibiotic-containing agar plates. Pure cultures of 308 isolates were obtained from Luria-Bertani (LB) agar plates (Nacalai Tesque Inc., Kyoto, Japan) without antibiotics. Bacterial genomic DNA was extracted by using the boiled lysate method as previously described (63). DNA was stored at −20°C prior to molecular screening.

### PCR detection of *mcr* genes and bacterial identification

All the isolates (308) were screened by PCR to identify the *mcr* genes (*mcr-1* to *mcr-9*), as previously described (6, 64). PCR was conducted in two multiplex assays with 2-µM dNTPs and 10-µM primer concentration using the Tks Gflex DNA polymerase (Takara Bio Inc., Shiga, Japan). The PCR amplicons were subjected to gel electrophoresis in a 3% agarose gel, followed by ethidium bromide staining and visualization under ultraviolet light. Additionally, the full-length *mcr-9* gene was amplified using the primers MCR9-WGF (5′-ATGCCTGTACTTTTCAGGGTG-3′) and MCR9-WGR (5′-TTAGCCACGGCATTCGA-3′). The PCR products were treated with ExoSAP-IT (Thermo Fisher Scientific) and submitted for Sanger sequencing by Eurofins Genomics, Japan. Bacterial identification was performed by amplifying the 16S rRNA gene using the universal primers 27F (5′-AGAGTTTGATCMTGGCTCAG-3′) and 1492R (5′-CGGYTACCTTGTTACGACTT-3′).

### Molecular screening for other antimicrobial resistance genes

Two isolates harbored the *mcr-9* genotype. Those isolates were further screened using a newly developed eicosaplex/octaplex PCR system (65) to identify β-lactamase encoding genes (*bla*_SHV_, *bla*_TEM_, *bla*_CTX-M_, *bla*_OXA-1_, *bla*_OXA-2_, *bla*_OXA-5_, *bla*_OXA-9_, and plasmid-mediated AmpC β-lactamases), integrons (class 1, class 2, and class 3), and the aminoglycoside acetyltransferase gene [*aac(6′)-Ib*]. PCR detection of acquired carbapenemases was also investigated (66).

### Antimicrobial susceptibility testing

The MICs of 12 antimicrobial agents were determined by using the standardized broth microdilution method according to the interpretative criteria described by the Clinical and Laboratory Standards Institute (CLSI 2020) (67). The breakpoint for colistin was determined according to the European Committee on Antimicrobial Susceptibility Testing (EUCAST 2021) (68). Antimicrobial agents included ampicillin, cefotaxime, ceftazidime, cefoxitin, meropenem, kanamycin, gentamicin, chloramphenicol, ciprofloxacin, tetracycline, colistin, and fosfomycin. A stock solution of each antimicrobial agent was prepared and diluted according to the CLSI recommendations. Quality control for MIC analysis was performed using *E. coli* American Type Culture Collection (ATCC) 25922.

### Whole genome sequencing

Complete genome sequencing was performed by using next-generation sequencing (NGS) platforms. Total bacterial genomic DNA was prepared using a Qiagen Genomic-tip 20/G kit (Qiagen) in accordance with the manufacturer’s guidelines. DNA sequencing libraries for Illumina MiSeq (short reads) were prepared using an Enzymatic 5× WGS fragmentation mix and WGS ligase reagents (Qiagen, Hilden, Germany) following the manufacturer’s recommendations. For Oxford Nanopore sequencing, a library was prepared by using a Rapid Barcoding Sequencing Kit (SQK-RBK004) (Oxford Nanopore Technologies, Oxford, UK). Sequencing libraries were purified, loaded onto an FLO-MIN106 flow cell, and sequenced using a GridION device (Oxford Nanopore Technologies).

### Genome and bioinformatics analyses

A *de novo* hybrid assembly of all sequences (short reads and long reads) was performed using Unicycler software v0.4.8 (https://github.com/rrwick/Unicycler) and annotated with DFAST bioinformatic tool (69). We analyzed the WGSs of the isolates at the Center for Genomic Epidemiology (http://www.genomicepidemiology.org/services/). Bacterial species were predicted using the KmerFinder v3.2, based on the fast k-mer algorithm (https://cge.food.dtu.dk/services/KmerFinder/). The acquired antimicrobial resistance genes were identified using ResFinder v4.1 (https://cge.food.dtu.dk/services/ResFinder/). Plasmid incompatibility groups, mobile genetic elements, and multilocus sequence types (MLSTs) were identified using PlasmidFinder v2.0 (https://cge.food.dtu.dk/services/PlasmidFinder/), MobileElementFinder v1.0.3 (https://cge.cbs.dtu.dk/services/MobileElementFinder/), and the MLST tool v2.0 (https://cge.food.dtu.dk/services/MLST/), respectively. The SnapGene Viewer software v7.0 was used to visualize the genomes and the *mcr-9*-bearing plasmid sequences. Additionally, a comparative genome analysis was performed by using BLAST/BRIG (39) and JSpeciesWS (37). Furthermore, *in silico* DNA-DNA hybridization using GGDC (38) was used to measure the degree of similarity and correlation between *mcr-9*-bearing plasmids sequences.

### Conjugation assay

Transferability of *mcr-9*-bearing plasmids was determined by using filter-mating conjugation assays, as previously described (63). Exponential-phase lysogeny broth cultures of the donor and azide-resistant *E. coli* J53 recipient strains were used. Both the donor and recipient strains were mixed in the ratio 1:9 (100-µL donor:900-µL recipient) and centrifuged for 3 min at 6,000 rpm. The supernatant was removed, and the pellets were resuspended in 200-µL LB broth. The resulting suspension was then plated on a conjugation filter on LB agar and incubated for 3‒5 h at 37°C. Transconjugants were selected on LB agar containing 100-µg/mL sodium azide and 4-µg/mL colistin. Transconjugants were confirmed by PCR targeting the *mcr-9* gene and antimicrobial susceptibility testing. Unlike *E. cloacae* CST17-2, the *mcr-9*-bearing plasmid within *R. ornithinolytica* CST129-1 was not successfully transferred to *E. coli* J53, after two-time trials. We repeated the experiment at 25°C and 30°C, but conjugation failed again at these temperatures. We further used two different strains of colistin-susceptible (MIC ≤ 1 µg/mL) *R. ornithinolytica* (MA-1-1-r and CAA-1-5-r) as recipients for repeat conjugation at varying temperatures, although this was unsuccessful. Furthermore, a colistin-susceptible (MIC 0.125 µg/mL) *K. pneumoniae* strain LM-22-1 (accession number DRA009528) carrying *mcr-9* on a 281,251-bp plasmid of IncHI2 backbone was used as a donor to *R. ornithinolytica* (MA-1-1-r and CAA-1-5-r) because we hypothesized that *mcr-9* may be expressed by other proteins in *Raoultella* spp. In this context, conjugation was successful leading to *mcr-9* activation.

### PCR-based replicon typing

Plasmids were isolated from *mcr-9*-carrying isolates as well as the *E. coli* J53 transconjugant using the alkaline lysis method as previously described (70). PCR-based replicon typing was then performed targeting the IncHI2 plasmid incompatibility group as previously described (71).

### Sequence alignment of *mcr-9* proteins

From the plasmid sequences of *E. cloacae* CST17-2, *R. ornithinolytica* CST129-1, and *K pneumoniae* LM22-1, the nucleotide sequences of *mcr-9* were retrieved using the SnapGene genome visualizer and translated into amino acid sequence using the EMBOSS Transeq tool (https://www.ebi.ac.uk/Tools/st/emboss_transeq/). The translated protein sequences were then imported into ClustalX v2.1 and saved into a FASTA file format. Then, multiple protein sequence alignment was performed using Clustal Omega method available in SnapGene Viewer software v7.0.

## Data Availability

The nucleotide sequences of *mcr*-9-carrying *E. cloacae* CST17-2 and *R. ornithinolytica* CST129-1 described in this study were deposited in the DDBJ Sequence Read Archive (DRA) under accession numbers DRR424647-DRR424648 and DRR424649-DRR424650, respectively (BioSample SAMD00565468 and SAMD00565469 for *E. cloacae* CST17-2 and *R. ornithinolytica* CST129-1, respectively), under BioProject accession number PRJDB14934.

## SUPPLEMENTAL MATERIAL

Fig. S1 spectrum.01063-23-s0001.docx Amino acid sequence alignment of the *mcr*-9 genes.

ASM does not own the copyrights to Supplemental Material that may be linked to, or accessed through, an article. The authors have granted ASM a non-exclusive, world-wide license to publish the Supplemental Material files. Please contact the corresponding author directly for reuse.
