# Supplementary figures and images for: Emergence of colistin-resistant Enterobacter cloacae and Raoultella ornithinolytica carrying the phosphoethanolamine transferase gene, mcr-9, derived from vegetables in Japan
Source: Microbiol Spectr. 2023 Nov 1;11(6):e01063-23. doi: 10.1128/spectrum.01063-23 (PMC10714742; doi:10.1128/spectrum.01063-23)

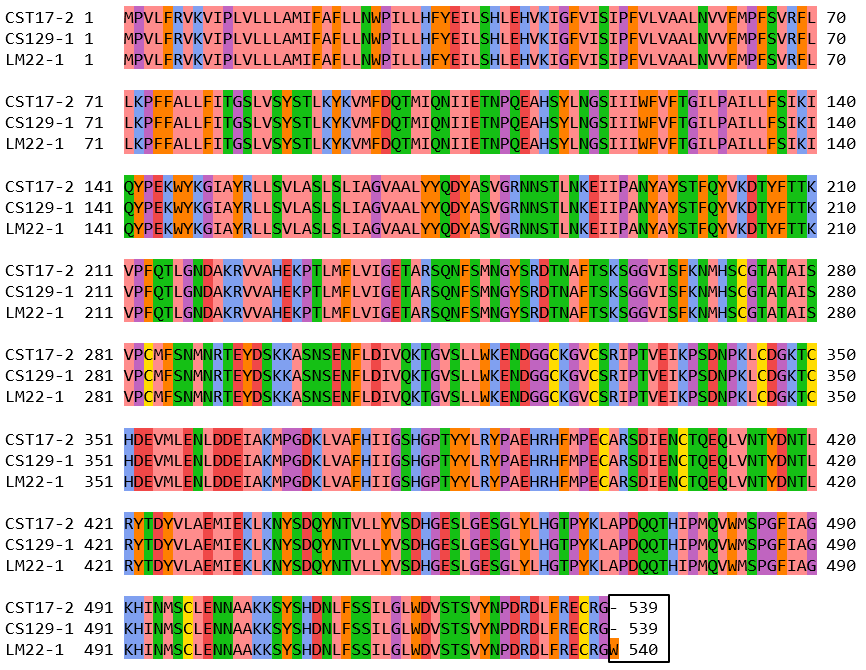


**Fig. S1** Amino acid sequence alignment of the *mcr-9* genes obtained from isolates used in this study.

Supplement: Fig. S1 — Amino acid sequence alignment of the mcr-9 genes. [file spectrum.01063-23-s0001.docx]
